# Thinking differently about lupus

**DOI:** 10.7554/eLife.15352

**Published:** 2016-03-29

**Authors:** Arthur Wuster, Timothy W Behrens

**Affiliations:** Genentech Inc., South San Francisco, United States; Genentech Inc., South San Francisco, United Statesbehrens.tim@gene.com

**Keywords:** targeted sequencing, SLE risk, haplotype, HLA, LD, risk allele, Human

## Abstract

A search for the genetic causes of an autoimmune disease called systemic lupus erythematosus reveals a new twist on an old story.

**Related research article** Raj P, Rai E, Song R, Khan S, Wakeland BE, Viswanathan K, Arana C, Liang C, Zhang B, Dozmorov I, Carr-Johnson F, Mitrovic M, Wiley GB, Kelly JA, Lauwerys BR, Olsen NJ, Cotsapas C, Garcia CK, Wise CA, Harley JB, Nath SK, James JA, Jacob CO, Tsao BP, Pasare C, Karp DR, Li QZ, Gaffney PM, Wakeland EK. 2016. Regulatory polymorphisms modulate the expression of HLA class II molecules and promote autoimmunity. *eLife* **5**:e12089. doi: 10.7554/eLife.12089

**Image** About half of people with SLE have a skin rash, often involving the cheeks and bridge of the nose (Credit: Doktorinternet)

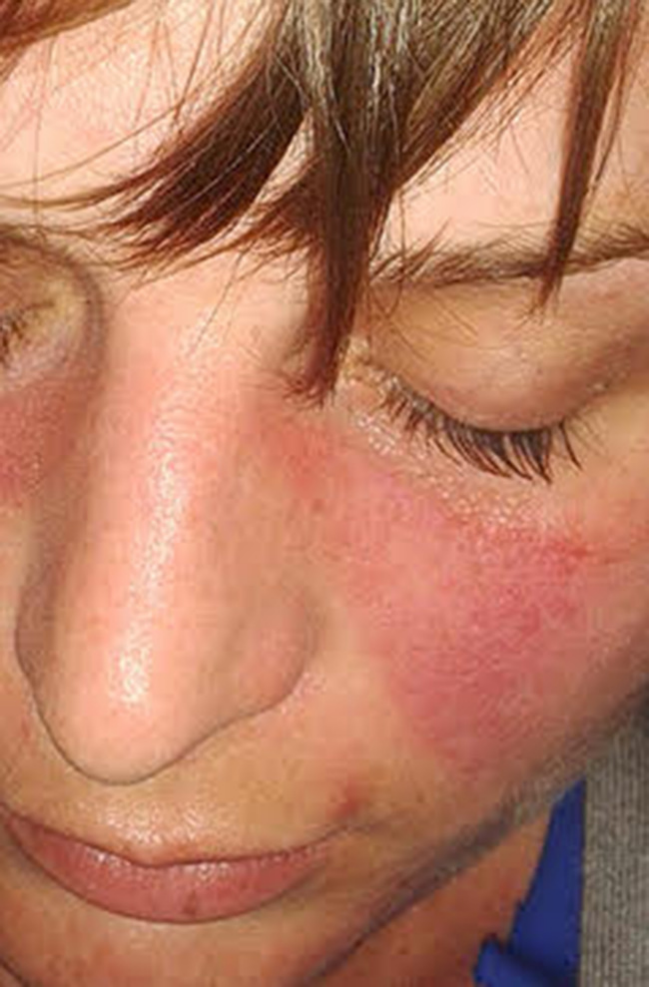

It is always exciting when a new study opens up a whole new way of thinking about a scientific problem. This is especially true if the problem is a health condition that is both poorly understood and incurable. Systemic lupus erythematosus (SLE) is an autoimmune disease in which the immune system attacks healthy tissue – including the skin, joints and many internal organs – by mistake. Moreover, the underlying causes of the disease are not fully understood, so available treatments only tackle its symptoms. Now, in eLife, Patrick Gaffney, Edward Wakeland and colleagues – who include Prithvi Raj and Ekta Rai as joint first authors – report an extensive sequencing study of over 1,000 people with SLE that provides many new insights into the genetics of lupus (Raj et al., 2016).

The researchers – who are based at the University of Texas Southwestern Medical Center, the Oklahoma Medical Research Foundation and other centers in the United States, Belgium and India – focused on 16 regions of the human genome that are known to influence a person’s risk of SLE. Their findings highlight the complexity of the changes in the genome that can predispose someone to develop SLE. Amongst the treasure-trove of data is one gold nugget that stands apart: the discovery that the expression level of the so-called Human Leukocyte Antigen (HLA) class II genes contributes to risk of the disease. This is an entirely new twist on an old story.

The HLA genes are found in a long stretch of DNA on chromosome 6 that contains more than 140 protein-coding genes, many of which are important for immune responses (MHC Sequencing Consortium, 1999; de Bakker et al., 2006). The class I genes encode proteins that are found on the outer membrane of all cells, whereas the class II genes encode proteins found only on specialized immune cells called antigen-presenting cells. In both cases, these proteins bind to small fragments of other proteins and then display them to the immune system. Class I proteins display fragments from within our own cells and protect us from cancer, viral infections and certain bacteria that can live within our cells. Class II proteins, on the other hand, display fragments from foreign invaders and are important for instructing the immune system to mount neutralizing responses to parasites and bacteria that live outside of cells.

The HLA class I and II genes are amongst the most variable DNA sequences in the human genome. There are more than 8,000 different variants, or alleles, of class I genes and more than 2,500 class II alleles (Robinson et al., 2015). These alleles encode slightly different proteins, each with the potential to bind to, and display, different protein fragments.

Hundreds of studies have previously linked class I and II variants with the risk of specific diseases and, without exception, these studies have emphasized the sequence diversity of class I and II genes as the important factor (Welter et al., 2014. For example, individuals who carry the class I allele known as B27 are strongly predisposed to developing a type of arthritis called ankylosing spondylitis, whereas people without the B27 allele appear to be protected against the disease (Caffrey and James, 1973).

The new findings show that the HLA class II alleles conferring risk for SLE encode proteins that are found in greater numbers on the surface of antigen-presenting cells than those encoded by alleles that do not increase the risk (Raj et al., 2016). These data suggest, for the first time, that the abundance of class II proteins on antigen-presenting cells could strongly influence risk of certain diseases.

It remains to be determined how differences in the abundance of class II molecules translate to increased disease risk. Higher levels of class II molecules on antigen-presenting cells could increase the chances that circulating immune cells bind to these molecules, and lead to more efficient immune and autoimmune responses. Another possibility is that the extra class II proteins on antigen-presenting cells could adversely affect a developmental process that would normally eliminate those immune cells that have the potential to cause autoimmune disorders.

Further studies now need to extend the analysis of Raj, Rai et al. to the other major class II alleles that are associated with disease in humans. It will also be important to see if this paradigm extends to the class I alleles. These studies will improve our understanding of the earliest events in autoimmunity and hopefully lead to new treatment options for autoimmune disorders.
